# The impact of the introduction of fidaxomicin on the management of *Clostridium difficile* infection in seven NHS secondary care hospitals in England: a series of local service evaluations

**DOI:** 10.1007/s10096-015-2538-z

**Published:** 2015-12-12

**Authors:** S. D. Goldenberg, S. Brown, L. Edwards, D. Gnanarajah, P. Howard, D. Jenkins, D. Nayar, M. Pasztor, S. Oliver, T. Planche, J. A. T. Sandoe, P. Wade, L. Whitney

**Affiliations:** Centre for Clinical Infection and Diagnostics Research, King’s College, London and Guy’s and St Thomas’ NHS Foundation Trust, 5th Floor, North Wing, St Thomas’ Hospital, Westminster Bridge Road, London, SE1 7EH UK; Department of Microbiology, County Durham and Darlington NHS Foundation Trust, Durham, UK; University Hospitals of Leicester NHS Trust, Leicester, UK; Derby Hospitals NHS Foundation Trust, Derby, UK; Department of Microbiology, University of Leeds and Leeds Teaching Hospitals NHS Trust, Leeds, UK; University Hospitals of Morecambe Bay NHS Foundation Trust, Lancaster, UK; pH Associates Ltd, Marlow, UK; St George’s Healthcare NHS Trust, London, UK

## Abstract

*Clostridium difficile* infection (CDI) is associated with high mortality. Reducing incidence is a priority for patients, clinicians, the National Health Service (NHS) and Public Health England alike. In June 2012, fidaxomicin (FDX) was launched for the treatment of adults with CDI. The objective of this evaluation was to collect robust real-world data to understand the effectiveness of FDX in routine practice. In seven hospitals introducing FDX between July 2012 and July 2013, data were collected retrospectively from medical records on CDI episodes occurring 12 months before/after the introduction of FDX. All hospitalised patients aged ≥18 years with primary CDI (diarrhoea with presence of toxin A/B without a previous CDI in the previous 3 months) were included. Recurrence was defined as in-patient diarrhoea re-emergence requiring treatment any time within 3 months after the first episode. Each hospital had a different protocol for the use of FDX. In hospitals A and B, where FDX was used first line for all primary and recurrent episodes, the recurrence rate reduced from 10.6 % to 3.1 % and from 16.3 % to 3.1 %, with a significant difference in 28-day mortality from 18.2 % to 3.1 % (*p* < 0.05) and 17.3 % to 6.3 % (*p* < 0.05) for hospitals A and B, respectively. In hospitals using FDX in selected patients only, the changes in recurrence rates and mortality were less marked. The pattern of adoption of FDX appears to affect its impact on CDI outcome, with maximum reduction in recurrence and all-cause mortality where it is used as first-line treatment.

## Introduction

*Clostridium difficile* infection (CDI) is a major public health concern [1] and is associated with prolonged hospital stay [2, 3], hospital readmissions [4, 5] and increased mortality [6–8]. Recurrence of disease is a significant problem, with 20–30 % of patients experiencing a recurrent episode following initial resolution [9, 10]. The European Society of Clinical Microbiology and Infectious Diseases (ESCMID) guidelines recognise that the high recurrence rate is among the most important problems posed by CDI [11]. In 2012, fidaxomicin (FDX) became available in the UK for the treatment of CDI. Two double-blind, randomised controlled trials (RCTs) showed non-inferiority of FDX compared to vancomycin with respect to initial CDI cure rates, and significantly lower recurrence rates in patients treated with FDX [12, 13]. Data on the real-world use of FDX outside of a clinical trial setting are lacking.

We report a series of local service evaluations conducted to evaluate the impact of FDX introduction on service delivery and CDI management in real-world conditions. Although conducted primarily to inform local decision-making, the results of the evaluations reported here detail differences in the experiences of routine FDX use in the English National Health Service (NHS).

## Materials and methods

A series of local service evaluations was conducted between December 2013 and October 2014 in collaboration with microbiologists, infection control clinicians and pharmacists in seven NHS hospitals in England, each offering a range of services, including: secondary, tertiary, acute, long-stay, elective and non-elective care. The primary objective of each evaluation was to describe CDI recurrence rates occurring in the year before (pre-) and after (post-) local introduction of FDX. Secondary objectives included describing: time to clinical cure of CDI; 28-day and 3-month all-cause mortality; CDI-related treatment pathways (including times from symptom onset to stool sampling, diagnosis and symptom resolution) and resource use [hospital length of stay (LOS), intensive care unit (ICU) admission rate and LOS, duration of treatment, readmission rate and LOS for both primary and recurrent episodes] in the year before and after FDX introduction; and characteristics of patients receiving FDX.

Hospitals were invited to conduct an evaluation based on interest in describing the impact of local FDX introduction and the availability of at least 20 cases of CDI per year to allow the generation of meaningful results locally. All hospitals conducting an evaluation commenced FDX use between July 2012 and July 2013.

Service evaluations are exempt from ethical review and patient consent in the UK [14]. Each hospital approved the evaluation protocol and release of fully anonymised patient data to pH Associates Ltd, a research consultancy company, for analysis.

Each evaluation was conducted according to a common protocol. Primary CDI was defined as diarrhoea with >3 consecutive unformed bowel movements and presence of *C. difficile* toxin A/B in stool (see Table 1 for the testing protocols). Two different definitions of CDI recurrence were used to accommodate local differences in repeating *C. difficile* toxin testing within 28 days of the primary infection. In hospitals A, C and G, policy was to define recurrence as symptomatic (i.e. reappearance of diarrhoea to an extent, judged by the frequency of passed unformed stools, that was greater than the frequency noted on the last day of medication) with treatment for CDI and toxin confirmed. In hospitals B, D, E and F, policy was to assume recurrence if patients were symptomatic and given treatment for CDI, without a requirement for toxin testing. CDI was considered to be community-acquired if the symptoms and/or sample for toxin testing were documented within 48 h of hospital admission.

**Table 1:** *Clostridium difficile* toxin testing policies and *C. difficile* infection (CDI) severity criteria

Hospital	*C. difficile* toxin testing policy	CDI severity criteria
A	Two-step testing method: 1. glutamate dehydrogenase (GDH) enzyme immunoassay (EIA); 2. *C. difficile* toxin EIA	According to the ESCMID definitions
B	Two-step testing method: 1. GDH EIA; 2. *C. difficile* toxin EIA	Severity parameters not collected for this evaluation
C	Two-step testing method: 1. GDH EIA; 2. *C. difficile* cytotoxin assay	Life-threatening: any of: hypotension (not responsive to fluid challenge), partial or complete ileus or toxic megacolon Severe: any of: raised WCC >15 × 10^9^/L., acute kidney injury (e.g. >50 % rise in serum creatinine above baseline), temperature of >38.5 °̊C, evidence of severe colitis (e.g. guarding, abdominal tenderness Non-severe: diarrhoea without features of severe or life-threatening infection
D	Two-step testing method: 1. GDH EIA; 2. *C. difficile* toxin EIA	According to PHE guidelines
E	Three-step testing method: 1. screen with GDH EIA; 2. all GDH-positives are then tested for *C .difficile* toxin EIA; 3. if GDH-positive and toxin-negative, PCR testing	According to PHE guidelines
F	Two-step testing method: 1. GDH EIA; 2. *C. difficile* toxin EIA GDH-positive, toxin-negative has another stool sample tested at least 24 h later	According to PHE guidelines
G	Three-step testing method: 1. screen with GDH EIA; 2. all GDH-positives are tested for *C. difficile* toxin EIA, 3. if GDH-positive and toxin-negative, PCR testing	According to PHE guidelines

Recurrences presenting to the same hospital within 3 months of the primary CDI episode (first toxin-positive stool sample) were recorded for the evaluation. Clinical cure was defined as three or fewer unformed stools on two consecutive days following treatment. The date of diarrhoea resolution was noted as recorded in clinical records; if this was unavailable, the date diarrhoea was last documented or the stop date of CDI medication in the clinical record was noted. Severity of CDI could not be defined objectively by retrospective review and was recorded as documented in the clinical record.

**Fig. 1:**
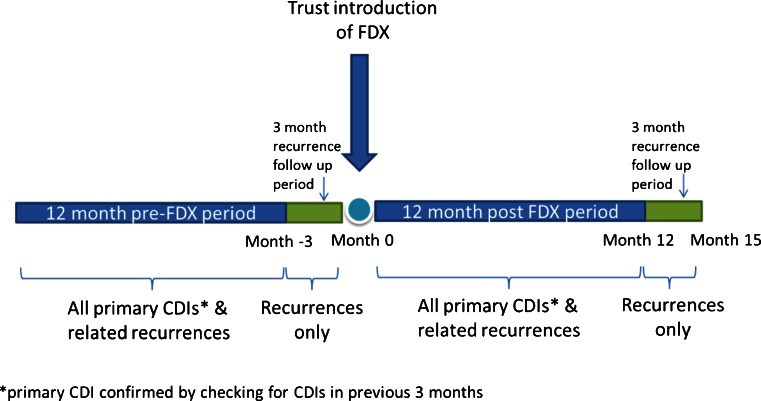
Service evaluation design showing two retrospective observation periods, before (pre) and after (post) introduction of fidaxomicin (FDX)

Each evaluation included two 12-month retrospective observation periods for primary CDI episodes, one before and one following local introduction of FDX. To ensure identification of all recurrences within 3 months of a primary episode, an additional 3-month observation period was included at the end of each observation period, for the collection of recurrence data only (Fig. 1). Similarly, microbiology records for the previous 3 months were checked for all CDI infections to verify a primary rather than recurrent episode.

All patients with a positive toxin A/B test for *C. difficile* within the evaluation period (pre- or post-FDX) were identified from microbiology department records. Patients aged <18 years were excluded, as were any cases that were diagnosed and managed in a community setting without hospital admission.

The full evaluation data were obtained for all episodes in the pre/post periods in all hospitals except hospital C. In this hospital, all episodes were included in the recurrence rate endpoint (Table 4, row 1) but the full dataset was collected in only a sample of patients in order to manage numbers (all other tables). The sample was taken by selecting the first seven episodes per month during the pre period and all electronically available episodes in the post period, both methods generating a random sample. Data were collected retrospectively from patients’ hospital records, including paper notes and electronic databases as appropriate in each hospital, using a standard data extraction process. Data were fully anonymised for analysis. The evaluation relied on data recorded routinely for patients’ clinical care. Analyses were conducted using the available data, with the number of patients included in each analysis stated where data were missing. Patients who died whilst inpatients were excluded from LOS calculations.

FDX was adopted for different uses in each hospital, with some using the agent first line in all patients (primary and recurrent CDI), others only for CDI recurrence and others only in selected patients (Table 2). In order to appreciate the response to different patterns of use, the results of each evaluation are presented separately, grouped according to the local policy for the use of FDX.

**Table 2:** Patterns of fidaxomicin (FDX) use

Pattern of FDX use		Hospital (total number of beds)						
		A (1100)	B (1000)	C^a^ (1806)	D (426)	E (890)	F (1187)	G (591)
		First line in all episodes	First line in all episodes	Used in selected episodes only^b^	First line for recurrence only	Used in selected episodes only^c^	Used in selected episodes only^d^	Used in selected episodes only^e^
Time period	Description of treatment	No. of primary episodes						
Pre-FDX	Non-FDX	66	98	79^a^	70	117	89	112
Post-FDX	Non-FDX	–	–	120	52	47	30	55
	FDX first line	27	62	10	1	17	52	6
	FDX not first line	5	2	10	3	29	7	8
	All treatments	32	64	140^a^	56	93	89	68
No. of recurrences								
Time period	Diagnostic approach	Clinical + toxin	Clinical	Clinical + toxin	Clinical	Clinical	Clinical	Clinical + toxin
Pre-FDX	Non-FDX	7	23	5^a^	5	22	16	9
Post-FDX	Non-FDX	–	–	16	–	7	6	2
	FDX first line	2	2	10	1	3	5	1
	FDX not first line	–	–	5	–	2	–	1
	All treatments	2	2	31^a^	1	12	11	4

^a^Data represent a sample of all episodes

^b^Recurrences and selected primary episodes according to microbiologist or infectious diseases consultant discretion

^c^All *C. difficile* toxin-positive patients except if: relative contraindication (age, severe renal failure, other medication), patient already recovered, patient discharged

^d^All *C. difficile* toxin-positive patients aged over 75 years old: first line; under 75 years old: for relapse and/or multiple co-morbidities and needing other antibiotics

^e^Recurrences and primary episodes in patients with risk factors that made a recurrence likely, e.g. immunosuppressed patients, those with several co-morbidities

**Fig. 2:**
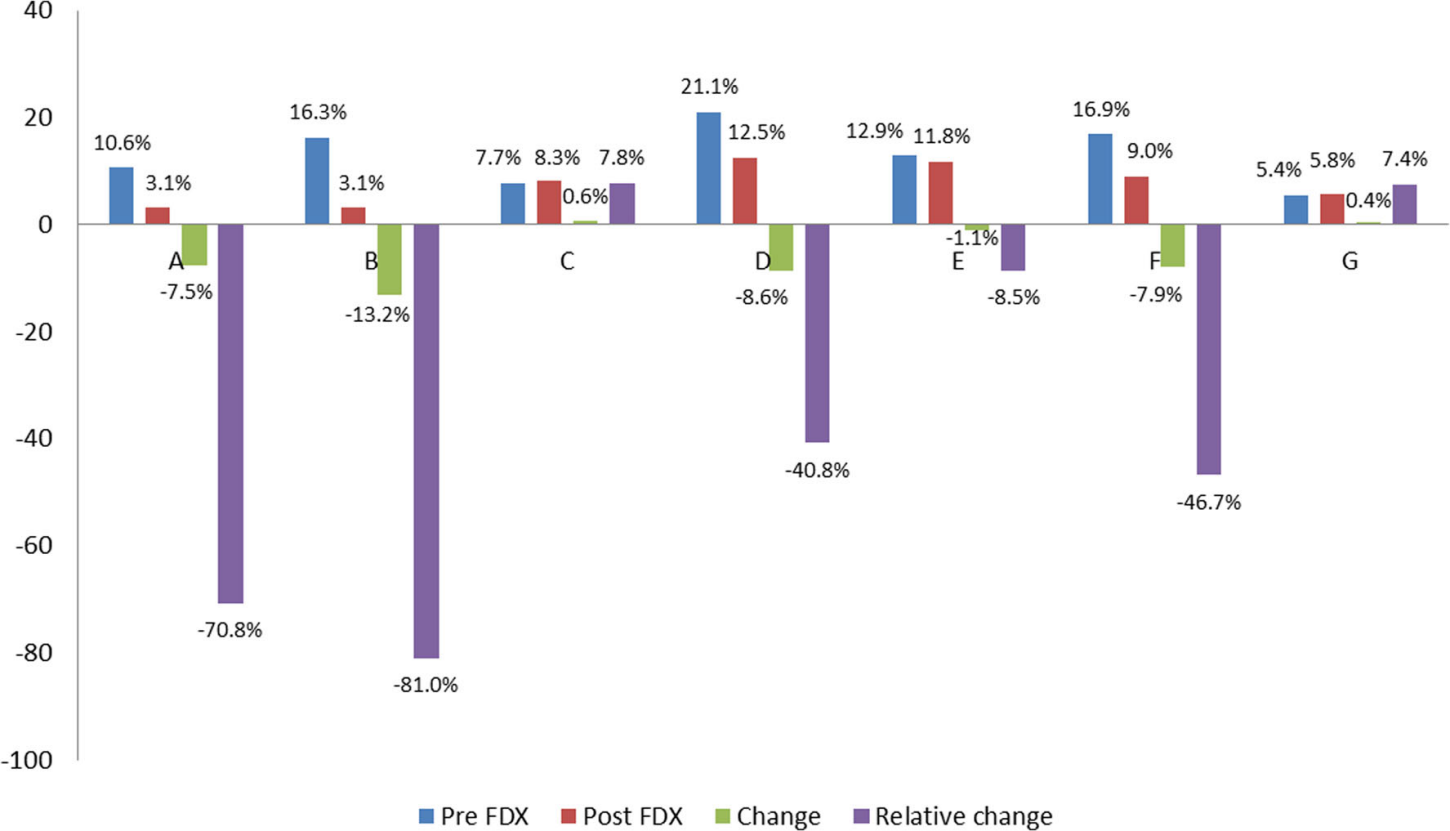
*Clostridium difficile* infection (CDI) recurrence rates in the pre- and post-FDX observation periods at participating hospitals

Values for each measure were tabulated and examined visually for possible differences between treatments (pre- and post-FDX) within each hospital. Differences between percentages or rates were tested for statistical significance using the Chi-squared (χ^2^) test, except when the total number of observations was too small and Fisher’s exact test was used. Continuous variables were compared using the unpaired *t*-test or Mann–Whitney *U*-test, depending on data distributions and homogeneity of the variance.

## Results

The FDX prescribing policy at each hospital is shown in Table 2. Following the introduction of FDX, hospitals A and B used FDX for all patients and hospital D used FDX first line in the single CDI recurrence but not for most primary CDI episodes. Hospitals C, E, F and G used FDX first line only in selected patients (primary and recurrent CDI).

The patient characteristics are shown in Table 3. The median age at primary and recurrent CDI ranged from 70 to 81 years and 43.5 to 84.5 years, respectively. Where severity was documented, the proportion of patients with primary CDI documented as severe/complicated ranged from 7.4 % to 72.2 %. Where documented, prior antibiotic use (within 3 months) ranged from 74.1 % to 98.1 % and proton pump inhibitor use concurrent with symptom onset from 0 % to 78.3 %. The proportion of patients hospitalised for ≥7 days before CDI ranged from 31.5 % to 58.9 % and the proportion with a prior stay in the ICU during the current hospital admission ranged from 0 % to 12.1 %. The proportion of community-acquired primary CDI ranged from 18.6 % to 44.6 %.

**Table 3:** Patient characteristics

	Hospital													
	A		B		C^d^		D		E		F		G	
	Pre	Post	Pre	Post	Pre	Post	Pre	Post	Pre	Post	Pre	Post	Pre	Post
Males with primary CDI, *n* (%)	38/66 (57.6%)	15/32 (46.9%)	52/98 (53.1%)	33/64 (51.6%)	30/79 (38.0%)	61/140 (43.6%)	32/70 (45.7%)	19/56 (33.9%)	64/116 (55.2%)	35/93 (37.6%), *p*=0.01	27/89 (30.3%)	35/89 (39.3%)	43/112 (38.4%)	31/69 (44.9%)
Median age at primary CDI (years)	70.0 (*n*=66)	77.5 (*n*=32)	77.0 (*n*=98)	76.0 (*n*=64)	78.0 (*n*=78)	74.0 (*n*=140), *p*=0.05	78.5 (*n*=70)	79.0 (*n*=56)	78.0 (*n*=112)	75.0 (*n*=93)	81.0 (*n*=89)	80.0 (*n*=89)	81.0 (*n*=112)	77.0 (*n*=69), *p*=0.05
Median age at recurrence (years)	82.0 (*n*=7)	43.5 (*n*=2), *p*=0.05	77.0 (*n*=17)	77.5 (*n*=2)	83.0 (*n*=5)	75.5 (*n*=30), *p*<0.05	83.0 (*n*=5)	83.0 (*n*=1)	69.0 (*n*=20)	70.0 (*n*=12)	82.0 (*n*=16)	80.0 (*n*=11)	78.0 (*n*=9)	84.5 (*n*=4)
Patients with severe/complicated primary CDI, *n* (%)^b^	15/55 (27.3%)	13/18 (72.2%), *p*<0.01	NA^a^	NA^a^	13/49 (26.5%)	35/88 (39.8%)	14/64 (21.9%)	11/51 (21.6%)	14/75 (18.7%)	5/31 (16.1%)	2/27 (7.4%)	11/64 (17.2%)	44/102 (43.1%)	34/65 (52.3%)
Patients with antibiotics before CDI (primary), *n* (%)^b^	47/58 (81.0%)	26/30 (86.7%)	NA^a^	NA^a^	62/74 (83.8%)	121/134 (90.3%)	53/63 (84.1%)	40/49 (81.6%)	83/112 (74.1%)	73/91 (80.2%)	68/80 (85.0%)	64/86 (74.4%)	80/87 (92.0%)	53/54 (98.1%)
Patients with PPI use (primary), *n* (%)^b^	32/57 (56.1%)	11/26 (42.3%)	NA^a^	NA^a^	47/60 (78.3%)	85/114 (74.6%)	38/57 (66.7%)	32/49 (65.3%)	53/115 (46.1%)	35/83 (42.2%)	0/46 (0.0%)	41/83 (49.4%), *p*<0.01	45/75 (60.0%)	27/49 (55.1%)
Patients with long stay^c^ in health care (primary), *n* (%)^b^	17/54 (31.5%)	11/27 (40.7%)	32/78 (41.0%)	31/54 (57.4%)	33/76 (43.4%)	57/133 (42.9%)	33/56 (58.9%)	22/53 (41.5%)	55/115 (47.8%)	33/93 (35.5%)	42/89 (47.2%)	35/88 (39.8%)	36/108 (33.3%)	20/50 (40.0%)
Patients with pre-CDI ICU stay (primary), *n* (%)^b^	8/66 (12.1%)	5/32 (15.6%)	NA^a^	NA^a^	3/79 (3.8%)	10/139 (7.2%)	5/70 (7.1%)	2/56 (3.6%)	6/115 (5.2%)	2/93 (2.2%)	1/86 (1.2%)	0/89 (0.0%)	0/112 (0.0%)	3/69 (4.3%), *p*<0.05
Patients with community-acquired CDI (primary), *n* (%)^b^	21/66 (31.8%)	14/32 (43.8%)	34/98 (34.7%)	18/64 (28.1%)	23/78 (29.5%)	41/140 (29.3%)	13/70 (18.6%)	15/56 (26.8%)	28/112 (25.0%)	30/93 (32.3%)	36/89 (40.4%)	25/89 (28.1%)	50/112 (44.6%)	27/69 (39.1%)

^a^Not available

^b^Numbers refer to CDI episodes

^c^Seven days or longer

^d^Data from a sample of all episodes

Table 4 shows the clinical outcomes at each hospital in the pre- and post-FDX periods. The recurrence rate from primary episodes of CDI significantly reduced in the post-FDX period in hospital B by 81.0 % (*p* = 0.01, see Fig. 2). Hospitals A, D, E and F had smaller, non-significant, relative reductions in recurrence rates and hospitals C and G had small non-significant relative increases in recurrence rates.

**Table 4:** Outcomes of treatment

	Hospital													
	A		B		C		D		E		F		G	
	Pre	Post	Pre	Post	Pre	Post	Pre	Post	Pre	Post	Pre	Post	Pre	Post
Recurrences/primary episodes (%)	7/66 (10.6%)	1/32 (3.1%)	16/98 (16.3%)	2/64 (3.1%)	19/246 (7.7%)	22/265 (8.3%)	15/71 (21.1%)	7/56 (12.5%)	15/116 (12.9%)	11/93 (11.8%)	15/89 (16.9%)	8/89 (9.0%)	6/112 (5.4%)	4/69 (5.8%)
% Change in recurrence rate (pre to post)	−7.5%		−13.2%		+0.6%		−8.6%		−1.1%		−7.9%		+0.4%	
Relative % change in recurrence rate	−70.1%		−81.0%, *p*=0.01		+7.8%		−40.8%		−8.5%		−46.7%		+7.4%	
Median time (days) to symptom resolution from diagnosis, primary episodes	7.5 (*n*=46)	13.0 (*n*=29), *p*<0.01	9.0 (*n*=92)	9.0 (*n*=64)	9.0 (*n*=45)	10.0 (*n*=87), *p*=0.01	8.0 (*n*=62)	7.0 (*n*=51)	9.0 (*n*=93)	10.0 (*n*=75)	10.0 (*n*=80)	10.0 (*n*=79)	11.0 (*n*=74)	11.0 (*n*=53)
Median LOS^a^ (days) from diagnosis, primary episodes	9.0 (*n*=54)	19.0 (*n*=28), *p*=0.01	14.0 (*n*=90)	19.0 (*n*=57)	11.0 (*n*=57)	9.5 (*n*=106)	13.0 (*n*=51)	15.0 (*n*=45)	11.0 (*n*=77)	12.0 (*n*=76)	14.5 (*n*=76)	16.0 (*n*=71)	13.0 (*n*=77)	15.0 (*n*=51)
ICU admission rate, following CDI diagnosis, primary episodes, *n* (%)	7/66 (10.6%)	2/32 (6.3%)	NK^b^	NK^b^	1/79 (1.3%)	2/140 (1.4%)	1/70 (1.4%)	1/56 (1.8%)	2/116 (1.7%)	3/93 (3.2%)	1/89 (1.1%)	0/89 (0.0%)	1/112 (0.9%)	0/69 (0.0%)
Readmission rate within 30 days of primary episode, *n* (%)	16/54 (29.6%)	2/27 (7.4%), *p*<0.05	NK^b^	NK^b^	10/58 (17.2%)	32/109 (29.4%)	10/51 (19.6%)	7/46 (15.2%)	9/83 (10.8%)	9/78 (11.5%)	16/76 (21.1%)	18/70 (25.7%)	19/78 (24.4%)	9/53 (17.0%)
Median LOS (days) from diagnosis with recurrence^c^	16.0 (*n*=5)	5.5 (*n*=2)	33.0 (*n*=14)	40.0 (*n*=2)	36.0 (*n*=5)	11.0 (*n*=25)	5.0 (*n*=3)	0 (N/A^d^)	15.0 (*n*=15)	8.0 (*n*=11)	14.5 (*n*=12)	23.0 (*n*=10)	9.0 (*n*=5)	27.0 (*n*=4)
Readmission rate within 30 days of recurrence^c^, *n* (%)	2/5 (40.0%)	0/2 (0.0%)	NK^b^	NK^b^	3/5 (60.0%)	11/25 (44.0%)	1/3 (33.3%)	0 (N/A^d^)	3/16 (18.8%)	2/11 (18.2%)	2/10 (20.0%)	0/8 (0.0%)	1/5 (20.0%)	0/4 (0.0%)
28-day all-cause mortality, primary episodes, *n* (%)	12/66 (18.2%)	1/32 (3.1%) *p*<0.05	17/98 (17.3%)	4/64 (6.3%), *p*<0.05	16/77 (20.8%)	23/138 (16.7%)	20/70 (28.6%)	5/55 (9.1%), *p*<0.05	25/109 (22.9%)	18/90 (20.0%)	13/89 (14.6%)	20/89 (22.5%)	34/112 (30.4%)	13/69 (18.8%)

^a^Length of stay

^b^Not known: data not provided

^c^LOS and 30-day readmission rate for recurrences are based on all recurrences, i.e. recurrences of primary CDI and further (second, third, fourth) recurrences

^d^Not applicable: *n*=0

Also shown in Table 4, the median time from diagnosis of primary CDI to diarrhoea resolution in the post-FDX period increased significantly in hospitals A and C, but remained similar in other hospitals. The median LOS for the primary CDI increased in the post-FDX period in hospital A (*p* = 0.01) and non-significantly in hospital B (*p* = 0.06), but remained similar in other hospitals. The readmission rate within 30 days of discharge after the primary CDI reduced in the post-FDX period in hospital A (*p* <0.05). There were no significant changes in any other hospital. The median LOS for recurrent CDI episodes showed no significant difference in any hospital between the pre-/post-FDX periods, although the number of recurrences was small. Rates of readmission within 30 days following recurrences showed a non-significant reduction in the post-FDX period in all five hospitals providing this data, although the numbers were small. All-cause mortality within 28 days of primary episodes reduced significantly in the post-FDX period in hospitals A, B and D, reduced non-significantly in hospitals C, E and G, and non-significantly increased in hospital F (Table 4).

No adverse events attributed to FDX had been documented in the medical records of any of the patients included in these evaluations.

## Discussion

This series of local service evaluations was conducted primarily for local use but, considered together, the results provide some useful insights on the real-world impact of different FDX adoption strategies.

As expected, patients with CDI were predominantly elderly, with a median age of at least 70 years across the hospitals. The severity of primary CDI was not recorded for all patients in some hospitals, providing an incomplete picture. The wide variation between hospitals in the proportion of patients with severe/complicated CDI also suggests that severity may not be assessed according to the same criteria in different hospitals. Variation in the proportion of patients with community-acquired CDI between hospitals could be partly a reflection of local outbreaks. It would be interesting to understand the impact of FDX on different ribotypes of *C. difficile* in the real world; however, this information was not and is not routinely documented in the normal practice of these hospitals; it may be the subject for potential further study with a prospective design.

Pre-FDX recurrence rates varied between hospitals (5.4–21.1 %) but were generally lower than commonly quoted expected recurrence rates of 20–30 % [15, 16]. It is difficult to compare the results of RCTs with real-world evaluations (RWE) because RCTs generally include highly selected patients in a controlled environment, with strict monitoring, while RWE do not; hence, the difference is most likely to be due to less rigorous surveillance for returning symptoms, and different testing and reporting requirements for recurrences outside an RCT.

The greatest reduction in relative recurrence rates (>70 %) was observed in the two hospitals that used FDX as first line in all patients with CDI. RCTs showed that FDX reduced recurrence rates compared with vancomycin [12, 13] and, therefore, the reduced recurrence in hospitals A and B are consistent with expectations, since all patients were treated with FDX in the post-FDX period. In contrast, the hospitals using FDX more selectively (e.g. only in the elderly or those with severe CDI) observed more modest reductions in relative recurrence rates. Conversely, two hospitals where FDX was used most selectively (chiefly for recurrences only) observed a non-significant increase in recurrence rates.

These data suggest that FDX is most effective when used in all patients with CDI, with no clear indication of patient sub-groups most likely to benefit. However, the recurrence rates in all hospitals, irrespective of the pattern of adoption of FDX (3.1–12.5 %), were lower than those seen in the pivotal clinical trials (12.7–15.4 %) [12, 13], despite a shorter window for recording recurrence in the trials (30 days) compared to the service evaluations (3 months). This may be explained by the implementation of other infection control measures in the intervening period or changing patterns of ribotypes causing disease and/or the follow-up for recurrence in these evaluations only during the hospital admission and any readmissions, missing CDI recurrences diagnosed in the community.

The time to resolution of diarrhoea was not positively affected by the introduction of FDX in most hospitals and, in fact, there was a statistically significant increase from 7.5 to 13.0 days in hospital A and from 9 to 10 days in hospital C. However, the resolution of symptoms was not well documented in patients’ clinical notes, with proxy measures for symptom resolution collected in the absence of definitive documentation, making it difficult to infer the impact of FDX on symptom resolution.

The LOS for primary and recurrent episodes of CDI was not significantly affected by the introduction of FDX in any hospitals, and, in fact, increased in all except one hospital. The factors affecting the LOS remain unclear but may reflect the well-recognised challenge of discharging patients (particularly the elderly) from hospital [17–19]. The increase in LOS was most marked in hospitals A and B. These hospitals also demonstrated a significant reduction in 28-day mortality in the post-FDX period. Whilst purely speculative, it is possible that the increased LOS may be related to improved survival, with patients who might previously have died and been excluded from the LOS analysis in the pre-FDX period now surviving but requiring extended care.

Mortality rates (28-day all-cause) following primary episodes of CDI were reduced to the lowest levels in hospitals A and B, where FDX was used first line in all patients, although there was also a statistically significant reduction in hospital D, where FDX was only used first line for CDI recurrences.

Readmission within 30 days of the primary or recurrent episode was not consistently affected by the introduction of FDX. Only hospital A, where FDX was introduced for all episodes of CDI, observed a statistically significant reduction in the 30-day readmission rate following primary CDI episodes, although a contributor to this could be the relatively long average LOS among the patients with CDI at this hospital, which may mitigate against readmission. Nevertheless, this reduction is consistent with the significant reduction in the recurrence rate (readmission data were not available for hospital B, which also demonstrated a substantial difference in recurrence).

### Limitations

Some limitations of this series of service evaluations have been described already in the discussion of each outcome measure. In addition, the differing retest policies of hospitals affected our ability to quantify the recurrence rate consistently across all the evaluations, by positive toxin test result, i.e. different definitions of ‘recurrence’ were used between hospitals and, so, the absolute recurrence rates should not be compared between hospitals. However, definitions of recurrence were consistent over time at each hospital, so the pre-FDX vs. post-FDX comparisons for each hospital are valid. The time to recurrence according to toxin result was affected by each hospital’s retest policy; no toxin-positive recurrence could occur within 14 (or 28) days of primary CDI in the hospitals with this policy; hence, this cannot be compared between hospitals. The time to recurrence analysis was also affected by widespread missing dates of recurrence. The definition of resolution of symptoms was not well defined and prone to great variation between hospitals. In the hospitals where toxin positivity was required for the diagnosis of a recurrence, the LOS associated with primary CDI could be an over-estimate due to their criteria for the diagnosis of recurrence. Some hospitals did not collect, or not consistently, data on readmissions. This, again, limited us to comparing between pre- and post-FDX introduction within each hospital, rather than comparing outcomes between hospitals. In hospital C, the selection of a sample of patients for collection of the full study dataset introduces the possibility of bias compared to the other hospitals where all episodes of CDI were included. However, random sampling should have minimised this risk.

## Conclusion

Although not designed as a formal comparison between hospitals, the results of these service evaluations suggest that the pattern of adoption of fidaxomicin (FDX) may affect the overall outcomes of *Clostridium difficile* infection (CDI) treatment achieved by a hospital. In practice, the benefits of the superior effect of FDX on the recurrence rate and 28-day mortality may be realised to the greatest extent where FDX is used as the routine first-line option for the treatment of CDI rather than in selected patients only. A policy of selective use of FDX only for recurrences had good outcomes in one hospital but was based on a small number of treated cases and requires a larger sample size for confirmation.
